# Effect of hygiene and medication on preweaning survival and growth of Djallonké sheep in Atacora, Benin

**DOI:** 10.1007/s11250-012-0183-4

**Published:** 2012-05-27

**Authors:** Sanni Y. Doko, Pamphile Degla, Gilbert O. Edoun, Roel H. Bosma

**Affiliations:** 1 Faculté d’Agronomy, Département Production Animales, Université de Parakou, BP 64 Parakou, Benin; 2 Faculté d’Agronomy, Département d’Economie et de Sociologie Rurales, Université de Parakou, BP 64 Parakou, Benin; 3 Department Animal Sciences, Wageningen University, P.O. Box 338, 6700 AH Wageningen, The Netherlands

**Keywords:** Lamb, Housing, Mortality, Gain, Innovation, Livestock

## Abstract

Low sheep productivity in North Benin hampers economic development, and improvement can contribute to reduction of rural poverty and food insecurity. To reduce one of the constraints to the productivity of Djallonké sheep, high mortality during suckling in full rainy and start dry season, we tested hygienic measures and medication in improved housing. The effect of the two treatments and their combination on lamb performance and the internal rate of return (IRR) were compared to a control. For each treatment, survival and growth were observed in 20 lambs, living in 38 herds. Good housing and hygiene (daily cleaning and fortnightly disinfection of stable and water and feed troughs) reduced mortality and increased growth of suckling lambs until 3 months. Good housing plus medication (unique injection of vitamins and amino acids and with ivermectine for deworming, weekly tick treatment by spraying, and in case of diarrhea, antibiotic treatment) reduced mortality, but growth was not higher than the control. Accounting labor opportunity, the IRR was about equal for both, but capital investment was lower for the hygiene treatment which is thus more accessible to poor farmers. The combination of both treatments increased growth and benefits compared with the hygiene treatment, but decreased the IRR.

## Introduction

**Fig 1:**
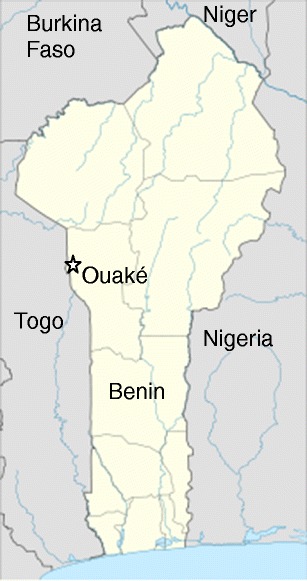
The location of Ouaké in Benin, West Africa

Low productivity of sheep was one of the constraints identified during a participatory assessment in Ouaké commune, just south of the Atacora in northwest Benin (Fig. 1). Most farmers raise Djallonké or related breeds of average-sized sheep found in the Sudanian zone of West and Central Africa under similar production conditions. The region has a high emigration rate due to the low productivity of agriculture, the main economic activity (Anonymous 2004). Such resource-poor regions are frequent in Africa and other continents; improved production of small ruminants can contribute to poverty alleviation and emigration reduction in such regions (Krishna 2010).

A participatory assessment of the sheep production system in six villages of the Atacora in Benin, had identified four interventions that might improve herd productivity (Soulemana 2009). One of the four proposed interventions aimed to reduce suckling lamb mortality in the rainy season through improved housing and hygiene. Developing sustainable practices requires cost–benefit analysis under farm conditions (Aw-Hassan 2008). We designed an on-farm experiment to assess whether the performance of suckling lambs can be improved cost-effectively: (1) through improved hygiene alone, (2) with only medicines (injections of vitamins and anthelmintics and spraying against ectoparasites), (3) through both hygiene and drugs, and (4) through hygiene and drugs plus curative treatment of diarrhea. Hereafter, we present materials and methods, the results, a discussion, and a conclusion.

## Materials and methods

Climate in Ouaké is characterized by dry and wet seasons of about 6 months with an average of 1,250 mm of rain (1970–1990, ASECNA station Djougou, in Anonymous 2004). The average temperature fluctuates between 29 and 33°C with maximum of 40°C in the dry hot season. The shallow arable soils are heavily degraded due to long cropping periods without fallow. Farmers hardly use manure or fertilizers; they extend cropping along river banks, and if done, they plow by using the wrong technique (Anonymous 2004). Most common crops are sorghum, cassava, peanut, and cowpea. Crop farmers keep livestock, cattle, sheep, goat, and poultry to complement family needs.

### Treatments

To test the hypothesis, we carried out a protocol with four treatments in the rainy season between June and October (Table 1). All animals were allowed to graze for 8 h, mostly starting at 7 am; they were tethered and moved once daily. Around noon, they were conducted to a water surface to drink; upon their return at the homestead, they were also given water. The animals in the control group did not receive any preventive or curative treatment. In case of diarrhea, the animals of the other three groups were treated with drugs. The first treatment focused on hygiene, the second, on medicinal prevention, and the third combined both. We provided chemicals, medicines, a bucket, and a broom for free.

**Table 1:** The four treatments of the experiment

Lot no.	Name	Treatment
0	Control	No disinfection and treatment next to customary interventions
1	Hygiene	Daily cleaning of stable, and water and feed troughs
		Fortnightly disinfection with a solution of cresyl (25 ml/l) of housing (floor and wall), troughs, and other tools
2	Medicines	Day 4: 2 ml stress-Vitam (mix of vitamins and amino acids) by intramuscular injection for each lamb^a^
		Day 7: deworming of all animals by subcutaneous injection of 0.1 ml/kg BW of Ivermectine^b^
		Weekly: tick treatment by spraying 2 ml/l of abotik (12.5 mg of amitraz/100 ml)^c^
		Curative: in case of diarrhea, one dose of veto-anti-diar (sulfaguanidine) in 0.5 l water. If persistent, intramuscular injection of oxycline 20 % (oxytetracycline) at 0.1 ml/kg BW
3	Hygiene and medicines	Both treatments 1 and 2 as mentioned above

*BW* body weight

^a^This is locally a veterinarian practice, but perhaps not recommendable for animals of this age

^b^For the lambs, this would have been superfluous

^c^Ivermectine is supposed to prevent tick-borne diseases, thus spraying was perhaps superfluous

### Animals

Each group was composed of 20 lambs (having less than 3 days) divided over 38 herds, i.e., on average, a herd contained about 2 lambs only. The herds contained a total of 70 ewes. The herds were from 13 different villages in six districts of Ouaké commune. We used the following criteria to choose the herds: number of ewes close to delivery, number of lambs younger than 1 week, and availability and experience of the owner. The herds were owned by crop farmers (20), traders (14), and pastoralists (4), who kept the lambs under quite similar conditions. The majority of the caretakers were women.

At night, the animals of the experimental groups were housed, either in a round hut in clay with open windows for ventilation or in an enclosure between huts, both having a straw roof. Most farmers provided litter from peanut straw.

The first 3–4 days after delivery of the lamb, the ewe is fed at home. The next 7–10 days, the ewe joins the other animals to the pasture, while the lamb stays at home where it may receive water and cooking remnants. After about 2 weeks, the lamb joins its mother on the pasture. Especially, day 3 to 14 is stressful for the lamb, and local veterinarian practice advices intramuscularly injection of stress-Vitam (a complex of vitamins and amino acids produced by Vétoquinol) in case of undernutrition.

Under prevalent housing conditions, diarrhea in lambs is mostly associated with coccidia. Therefore, animals showing diarrhea were first treated with Sulfaguanidine.

### Data collection and analysis

We weighed the lambs within 3 days after birth and every fortnight after birth until they were 92 days, between 7 and 9 a.m. before the herd was taken to graze. The scale of 5 kg, used to weigh the young lambs, had a precision of 50 g, and the scale of 20 kg had a precision of 200 g. We registered weights, disease prevalence and type, treatments and mortality on individual datasheets, and collected information on the owner and the herd (composition and management) in a separate survey. We encoded the data in a MS Excel^®^ datasheet and did statistical analysis by using one-way ANOVA from SPSS^®^ 16. The mortality rate was calculated in MS Excel as: ${1}00\, \times \,\left( {{{\text{number of dead lambs}} \left/ {\text{number of lambs born alive}} \right.}} \right)$.

To quantify labor cost, we monitored the number of hours that men, women, and youngsters from 6 to 14 years spent on feeding, herding, cleaning, and collecting feed. Total labor was calculated in ${\text{adult}}\,{\text{man}}\,{\text{equivalent}} = \left( {{\text{hour}}\,{\text{by}}\,{\text{man}}} \right) + 0.{75}\, \times \,\left( {{\text{hour}}\,{\text{by}}\,{\text{women}}} \right) + 0.{5}\, \times \,\left( {{\text{hour}}\,{\text{by}}\,{\text{youngsters}}\,{\text{of}}\,{6}--{14}\,{\text{years}}} \right)$(Norman 1972). One working day was assumed to be 8 h. A labor day in livestock keeping was paid at 605 Fcfa and in cropping between 900 and 1,200 Fcfa. We assumed an opportunity cost of 850 Fcfa/day especially as women and youngsters invested most time (see “Results”).

The value of the production was estimated by multiplying the weight gain with the average market price of a 3-month-old sheep expressed in Fcfa per kilogram. These lambs were sold for prices which varied between 5,000 and 10,000 Fcfa; these lambs weighed between 4.5 and 10.5 kg. Adults were sold at around 1,000 Fcfa/kg body weight; butchered meat was sold at 1,700 Fcfa/kg with bones and at 2,000 Fcfa/kg without bones. We assumed the value of the production to be 1,000 Fcfa/kg body weight (Gbedonou 2010).

Operational cost included salt, various feeds stuffs, salt, cresyl, oxycline 20 %, Ivermectrine 1 %, stress-Vitam, veto-anti-diar (sulfaguanidine), oxycline 20 % (oxytetracycline), and abotik (an insecticide for ticks). For all these, the total consumed quantity was multiplied by the unit cost. The cost of injecting was 105 Fcfa per animal per time.

The economic parameters were calculated with the following common formula:

$$ {\text{Total}}\,{\text{cost}} = {\text{operational}}\,{\text{cost}} + {\text{labor}}\,{\text{cost}} + {\text{fixed}}\,{\text{cost}} $$

$$ {\text{Gross}}\,{\text{margin}} = {\text{total}}\,{\text{value}}\,{\text{of}}\,{\text{production}} - \left( {{\text{operational cost}} + {\text{labor cost}}} \right) $$

$$ {\text{Net}}\,{\text{margin}} = {\text{gross}}\,{\text{margin}} - {\text{fixed}}\,{\text{cost}} $$

and

$$ {\text{Rate}}\,{\text{of}}\,{\text{return}} = {{{{\text{net}}\,{\text{margin}}}} \left/ {{{\text{total}}\,{\text{cost}}}} \right.}\, $$

To assess if the extra investment would be worthwhile for farmers, we calculated an internal rate of return: ${{{\left( {{\text{net}}\,{\text{margin}}\,\left( {1} \right) - {\text{net}}\,{\text{margin}}\,(0)} \right)\,}} \left/ {{\,\left( {{\text{total}}\,{\text{cost}}\,\left( {1} \right) - {\text{total}}\,{\text{cost}}\,(0)} \right)}} \right.}\,{ }$

## Results

The average mortality rate of lambs was close to 14 %. Mortality was highest in treatment 4, followed by treatment 2 (Table 2). Treatments 1 and 3 each lost one lamb. All casualties fell in the first month, and most of these lambs died in the first 2 weeks after birth (80 %). Most casualties were among the animals that were lightest at birth.

**Table 2:** The weight development (average ± standard deviation) and the mortality of the four treatments and the overall average (mean) in rainy season

Treatment	Mortality		Weight (kg)			
	(%)	n	Birth (20)	30 days	60 days	90 days
Control	30	14	1.45 ± 0.28	2.31 ± 0.48^a^	4.1 ± 0.4^a^	5.2 ± 0.5^a^
Hygiene	5	19	1.46 ± 0.50	3.25 ± 0.84^b^	5.6 ± 0.9^b^	7.9 ± 0.9^b^
Medicines (drugs)	15	17	1.44 ± 0.46	2.61 ± 0.41^a^	4.8 ± 0.3^a^	6.6 ± 0.6^a^
Hygiene and drugs	5	19	1.49 ± 0.38	3.81 ± 0.51^c^	6.6 ± 0.6^c^	9.3 ± 0.9^c^
Mean	14	69	1.46 ± 0.46	3.08 ± 0.82	5.4 ± 1.1	7.3 ± 1.8

Weights in the same column having a different superscript letter differ significantly (*P* < 0.05)

Diarrhea prevailed in all four treatments, and prevalence was 30, 25, 20, and 15 %, for ‘control’, ‘hygiene’, ‘medicines only’, and ‘hygiene and medicines’, respectively. Other prevalent diseases observed in the control and the medicinal prevention groups were only: mycotic dermatitis (6 %), pneumonia (2.5 %), foot rot (6 %), and scab (2.5 %). Prevalence of dermatitis and foot rot was slightly higher in the control (30 %) compared with those animals given the drug treatment (10 %). One ewe in treatment 3 suffered from mastitis and refused suckling.

The average birth weights were identical between treatments (Table 2). The weight development of the animals receiving both ‘hygiene and medicine’ was significantly better than that of the other three treatments. The weight gain of the ‘medicines’ only treatment was not significantly better than that in the ‘control’, while gain was significantly better in both treatments with hygiene.

During the 92 days of the experimental period, the farmers spent on average 5.7, 13.5, 9.4, and 17.3 days on herd maintenance for ‘control’, ‘hygiene’, ‘medicines only’, and ‘hygiene and medicines’, respectively. According to the nonweighted average, men invested half the time youngsters did, but women invested the most time (Table 3).

**Table 3:** The distribution of labor input (percent) for four sheep-maintenance activities by three gender groups

Type of activity	Women	Men	Youngsters
Feeding	50	18	32
Herding	13	11	77
Cleaning	52	14	34
Buying feed	77	23	0
Nonweighted average	48	17	36

The net margins of ‘hygiene’ and if ‘hygiene and medicines’ were significantly higher (*P* < 0.01) than that in the ‘control’ (Table 4). The net margin between ‘control’ and ‘medicines’ as well as that between ‘hygiene’ and ‘hygiene and medicines’ were not statistically different. However, due to the high operational cost and labor investment, the IRR of the treatments was not better than that in the ‘control’ (Table 4). The return on capital and labor invested remained best for the ‘control’, though not statistically. The marginal rate of return on the supplementary investment was about the same for the ‘hygiene’ compared with that of the ‘medicines only’ treatment, which was higher than that for the ‘hygiene and medicines’ treatment.

**Table 4:** Financial results of the four treatments for the herds (amounts in FCFA)

	Treatment			
Economic parameter	Control	Hygiene	Medicines	Hygiene and medicines
Gross income	52,500	115,920	87,720	140,580
Operational cost	2,070	9,916	7,267	18,401
Labor opportunity cost	4,830	11,500	8,021	14,663
Fixed cost	1,937	1,525	1,779	2,454
Gross margin	45,600	94,504	72,432	107,517
Net margin	43,663	92,979	70,653	105,063
Internal rate of return (%)	4.94	4.89	4.14	3.60
Marginal rate of return (%)		1.63	1.58	1.31

## Discussion

Notwithstanding, the remaining differences in husbandry practices between the herds, the treatments show significant effects. For farmers having labor available, the cleaning and disinfection of the housing are an efficient means to improve sheep performances in rainy season if their herd size is important enough. Also, weight gain is significantly better compared with the use of medicines only, which is promising for the other animals in the herd in case of the ‘hygiene’ treatment, but does not benefit other animals in case of the ‘medicines’ treatment. The societal benefit of the more efficient use of natural resources is not accounted for in the higher growth rate of the ‘hygiene’ and the ‘hygiene and medicines’ treatments, compared with that derived from the ‘medicines’ treatment and the ‘control’. The results show that common veterinary practice is prohibitive and provides a clear message for extension services: Drugs cannot replace hygiene. Institutional and other conditions need to be fulfilled before these messages can effectively reach farmers (Iniguez 2011).

Weights at 3 months of the best performing treatment were close to that found by Strutz and Glombitza (1986) and by Osaer et al. (2000): 9.0 and 8.6 kg, respectively. The weights we observed at 30, 60, and 90 days were within the range observed on station (Gbangboché et al. 2005, 2006), but slightly lower compared with those measured in Banikoara, Benin, between March and May: 5.3 ± 1.0, 7.7 ± 1.4, and 10.6 ± 1.9 kg, respectively (Youssao et al. 2008). This last could be due to the period; compared with the wet season, in dry season, animals are less stressed by parasites, and nutrition is not restricted by attaching. During free roaming in dry season, intake can be restricted, but the quality of feed selected can be good for small ruminants. This tendency was confirmed by both Bosma et al. (1996a, pp. 49–58) and Clément et al. (1997). In the subhumid zone of Mali, differences in weight gain of lambs born in different semesters are more pronounced after 60 days, when milk availability is reduced and lamb's nutritional requirements are increased (Bosma et al. 1996a, pp. 49–58; Bosma et al. 1996b).

The average death rate until 3 months is only half of the average found by Osaer et al. (2000) in Gambia. The relative high death rate in the control and in the drug treatment is probably due to the lack of hygiene, as animals are exposed to parasites (Gueye et al. 1994). The reduced weight gain in these treatments can be related to lack of hygiene through: (1) foot rot resulting in stress, lower intake, and even death (Lafia 1982); (2) pneumonia causing infections, fever, stress, and even death; (3) infection of the umbilical cord causing stress and followed by death for the young lambs. The difference between these two treatments in disease prevalence and death is due to the drug being used. In the control group, ticks and lice may reduce the growth of animals directly (Stachurski et al. 1988) and indirectly through the transmitted disease agents (Akimboade 1983).

Diarrhea was observed in all four groups, which was probably related to the early hour the animals were brought to the pasture. During humid season, the grasses are humid, and ticks and worms remain present until halfway the morning or even later after an early morning rain. The grazing hours should be adjusted to the humidity of the grasses, also because humidity reduces ingestion. To confirm the suspected cause of death, parasite levels of the treatments should have been monitored, and postmortem analysis should have been done; both were difficult under field conditions and available resources.

At least two aspects still restrict the productivity of the sheep: the low prolificacy in the wet season and the low weight at birth. A low weight at birth results in higher mortality of young suckling lambs (Armbruster 1991). A next step in the improvement would be to complement the ewes before mating for flushing and in the last 2 months of pregnancy to stimulate fetal growth.

## Conclusions

The results show that common veterinary practice is prohibitive and provides a clear message for extension services: Drugs cannot replace hygiene. Daily cleaning and fortnightly disinfection of housing and troughs reduced morbidity and mortality and improved growth rate of suckling lambs until 3 months. Medication only reduced mortality, but growth was not significantly higher compared with that of the control. Cost efficiency was about equal for both, taking into account labor opportunity cost. Combining these two treatments improved benefits, but decreased cost efficiency. Following Bosma et al. (1999), next step in this research would be to verify the effects of complementing ewes in the dry season before mating on prolificacy and before delivery on the lamb' birth weight.

## References

[CR1] Akimboade AP (1983). Experimental transmission of *Babesia bigemina* in sheep using infective tick of *Boophilus decoloratus*. Revue d'Elevage et de Médicine Vétérinaire des Pays Tropicaux.

[CR2] Anonymous, 2004. Plan de Développement Communal, Ouaké, (unpublished, Commune de Ouaké)

[CR3] Armbruster T, Peters KJ, Hadji-Thomas A (1991). Sheep production in the humid zone of West Africa, 3: Mortality and productivity of sheep in improved production systems in Ivory Coast. Journal of Animal Breeding and Genetics.

[CR4] Aw-Hassan AA (2008). Strategies for out-scaling participatory research approaches for sustaining agricultural research impacts. Development Practice.

[CR5] Bosma, R.H., Bengaly, K., Traoré, M. and Roeleveld, A., 1996a. L'élevage en voie d'intensification. Synthèse de la recherche sur les ruminants dans les exploitations agricoles mixtes au Mali-sud, (KIT Amsterdam)

[CR6] Bosma, R.H., Bengaly, K., Meurs, M., Diabaté, D., Sanogo, B. and Bagayogo, S., 1996b. The rôle of monitoring cattle and small ruminant productivity in livestock diagnostic studies, Southern Mali, In: Roeleveld A.C.W. and van den Broek, A.,: Focusing livestock system research. KIT-press, Amsterdam

[CR7] Bosma, R.H., Serme, R. and Zongo, L.R., 1999. La production ovine dans les provinces du Bulkiemdé et du Sanguié (Burkina Faso): diagnostic participatif et expérimentation paysanne, In: Renard G., Krieg S., Lawrence, P. and von Oppen, M.,: Farmers and scientist in a changing environment: addressing research in West Africa, (Proc. Conference 22–26 Février 1999, Cotonou, Bénin, Margraf), 479–489)

[CR8] Clément V, Poivey JP, Faugere O, Tillard E, Lancelot R, Gueye A, Richard D, Bibe B (1997). Etude de la variabilité des caractères de reproduction chez les petits ruminants en milieu traditionnel au Sénégal. Revue d'Elevage et de Médicine Vétérinaire des Pays Tropicaux.

[CR9] Gbangboche AB, Hornick JL, N'diaye MA, Edorh AP, Farnir F, Abiola FA, Leroy PL (2005). Caractérisation et maîtrise des paramètres de la reproduction et de la croissance des ovins djallonké (*Ovis amon**aries*). Annales Médecine Vététinaire.

[CR10] Gbangboche AB, Youssao AKI, Senou M, Adamou-N'diaye M, Ahissou A, Farnir F, Michaux C, Abiola FA, Leroy PL (2006). Examination of non-genetic factors affecting the growth performance of djallonke sheep in soudanian zone at the Okpara breeding farm of Benin. Tropical Animal Health and Production.

[CR11] Gbedonou, E.J., 2010. Analyse des rentabilités financière et économique des différents modes de conduite des agneuax Djallonké de présevrage dans la commune de Ouaké. Engineer graduation thesis, December 2009, Faculty of Agronomy, University of Parakou, Benin

[CR12] Gueye A, Mbengue MB, Diouf A (1994). Tiques et hémoparasites du bétail au Sénégal VI. La zone soudano-sahélienne. Revue d'Elevage et de Médicine Vétérinaire des Pays Tropicaux.

[CR13] Iniguez L (2011). The challenges of research and development of small ruminant production in dry areas. Small Ruminant Research.

[CR14] Krishna A (2010). Who became poor, who escaped poverty, and why? Developing and using a retrospective methodology in five countries, Journal of Policy Analyses and Management. Special Issue on Poverty Measurement.

[CR15] Lafia, S., 1982. Les tiques *Amblyommidae*, (unpublished PhD thesis, Université de Dakar)

[CR16] Norman DW (1972). An economic survey of three villages in Zaria province 2. Input-output study. Samaru miscellaneous papers 37.

[CR17] Osaer S, Goossens B, Eysker M, Geerts S (2000). The effects of prophylactic anthelmintic treatment on the productivity of traditionally managed Djallonke sheep and West African Dwarf goats kept under high trypanosomosis risk. Acta Tropica.

[CR18] Soulemana, M.A., 2009. Diagnostic participatif de la productivité des troupeaux ovins Djallonké dans les commune de Gogounou et de Ouaké au Nord du Benin. Engineer graduation thesis December 2008, Faculty of Agronomy, University of Parakou, Benin

[CR19] Stachurski F, Barre N, Camus E (1988). Incidence d'une infestation naturelle par la tique *Amblyomma variegatum* sur la croissance des bovins et des caprins créoles. Revue d'Elevage et de Médicine Vétérinaire des Pays Tropicaux.

[CR20] Strutz C, Glombitza F (1986). Moutons Djallonké élevés par les villageois au Congo, peuvent-ils être sélectionnés pour l'augmentation du poids. Revue d'Elevage et de Médicine Vétérinaire des Pays Tropicaux.

[CR21] Youssao AKI, Farougou S, Koutinhouin BG, Biobagou G, Kora BD (2008). Aptitudes maternelles de la brebis Djallonké en élevage traditionnel dans la commune de Banikoara au Bénin. Revue d'Elevage et de Médicine Vétérinaire des Pays Tropicaux.

